# *Candida albicans*-Cell Interactions Activate Innate Immune Defense in Human Palate Epithelial Primary Cells via Nitric Oxide (NO) and β-Defensin 2 (hBD-2)

**DOI:** 10.3390/cells8070707

**Published:** 2019-07-12

**Authors:** Ana Regina Casaroto, Rafaela Alves da Silva, Samira Salmeron, Maria Lúcia Rubo de Rezende, Thiago José Dionísio, Carlos Ferreira dos Santos, Karen Henriette Pinke, Maria Fátima Guarizo Klingbeil, Priscila Aranda Salomão, Marcelo Milanda Ribeiro Lopes, Vanessa Soares Lara

**Affiliations:** 1 Department of Surgery, Stomatology, Pathology and Radiology, Bauru School of Dentistry, University of São Paulo, 17012-901 Bauru, SP, Brazil; 2 Department of Prosthodontics and Periodontology, Bauru School of Dentistry, University of São Paulo, 17012-901 Bauru, SP, Brazil; 3 Department of Biological Sciences, Bauru School of Dentistry, University of São Paulo, 17012-901 Bauru, SP, Brazil; 4 Department of Oral Pathology, School of Dentistry, University of São Paulo, 03178-200 São Paulo, SP, Brazil

**Keywords:** fungal biofilm, oral epithelium, apoptosis, β-defensin, epithelial defense

## Abstract

The presence of *Candida albicans* in the biofilm underlying the dental prosthesis is related to denture stomatitis (DS), an inflammatory reaction of the oral mucosa. The oral epithelium, a component of the innate immune response, has the ability to react to fungal invasion. In this study, we evaluated the in vitro effect of viable *C. albicans* on the apoptosis, nitric oxide (NO) production, and β-defensin 2 (*hBD-2*) expression and production of human palate epithelial cells (HPECs). We further determined whether or not these effects were correlated with fungal invasion of epithelial cells. Interaction between HPEC primary culture and *C. albicans* was obtained through either direct or indirect cell–cell contact with a supernatant from a hyphal fungus. We found that the hyphae supernatants were sufficient to induce slight HPEC apoptosis, which occurred prior to the activation of the specific mechanisms of epithelial defense. The epithelial defense responses were found to occur via NO and antimicrobial peptide hBD-2 production only during direct contact between *C. albicans* and HPECs and coincided with the fungus’s intraepithelial invasion. However, although the hBD-2 levels remained constant in the HPEC supernatants over time, the NO release and *hBD-2* gene expression were reduced at a later time (10 h), indicating that the epithelial defense capacity against the fungal invasion was not maintained in later phases. This aspect of the immune response was associated with increased epithelial invasion and apoptosis maintenance.

## 1. Introduction

Denture stomatitis (DS) is an inflammatory reaction of the oral mucosa underlying removable dental prostheses, mainly upper dentures [1,2]. Individuals in the 50–80 year age group most frequently suffer from DS, with greater involvement of the palatal mucosa [3]. Although it is a disease of multifactorial etiology, DS is strongly related to the fungus *Candida albicans*, which is commonly found in the biofilm that forms on the inner surface of acrylic dentures [1,2]. Systemic factors are also involved in the pathogenesis of DS, such as immune response depression [4,5], which is common in elderly people [6,7].

*C. albicans* is a commensal fungus constituent of the normal mucosal microbiota in humans [8]. However, under suitably predisposing conditions, it is able to cause several mucosal diseases, including DS. The morphological transition from yeasts to filamentous hyphae and the virulence that is associated with this transition are crucial for the pathogenicity of *C. albicans* [9]. The filamentous hyphae grow by stretching and have the ability to penetrate oral tissues. This morphology is thought to be responsible for the initial inflammatory response in DS [10].

Despite the damage that penetration of hyphae into cells causes, the epithelium attempts to combat fungal cell growth and invasion of tissue [8]. The oral epithelium functions as a passive mechanical barrier that resists microbial infection. In addition to passive resistance, the epithelial cells actively respond by initiating an inflammation process, secreting cytokines and chemokines to alert various cell types for the activation of innate and adaptive immune responses [4,5,11].

Focusing on the innate immune response, epithelial cells also produce antimicrobial molecules against pathogens to contribute to combating micro-organisms before the infection is established [4,5,10]. The antimicrobial peptide β-defensins (hBD) is secreted by keratinocytes of oral mucosa [12] and has been shown to present antifungal activity against *C. albicans* [13,14]. Furthermore, these peptides participate in the modulation of innate and adaptive immune responses against a range of oral pathogens [15]. Nitric oxide (NO), which is synthesized by a variety of cells, including keratinocytes, is also induced in response to microbial infection [16], and plays an important role in a host’s defense against *C. albicans* via direct cell destruction or anion formation [17]. Moreover, NO was shown to be a potent antimicrobial agent against oral infections [6,18].

There is a lack of information on the role of epithelial cells in oral candidiasis, including in DS. Previous studies used an oral epithelial cell line derived from well-differentiated oral carcinomas (SCC15, ATCC; TR146; FaDu) [10,19,20,21], the immortalized oral keratinocyte line OKF6/TERT2 [22,23] and immortalized keratinocytes from human skin (HaCaTs) [24,25]. We did not find any studies involving a primary culture of human palate epithelial cells (HPECs), or studies on the relationship between the epithelial aggravation that *C. albicans* causes and the epithelial defense responses against this micro-organism. The present study examines the direct and indirect effects of *C. albicans* on HPEC defense over time. To complement the primary HPEC analyses, we performed experiments with the immortalized human gingival epithelial cell line OBA-9 in those cases where no or a low response was detected in the primary cells. The results show that aggressive events, such as the fungus’s invasion of HPECs and the induction of apoptosis in epithelial cells, were correlated with epithelial defense responses by NO and β-defensin 2 (hBD-2) production. In addition, the responses of the HPEC primary culture were found to be different from those of the immortalized keratinocyte cell line OBA-9, for example in terms of NO release.

## 2. Materials and Methods

### 2.1. Ethics Statement

Palatal mucosa samples (5 mm^2^) were biopsied from gingival graft patients after they had given written informed consent in compliance with the National Council of Health and Ethics Committee guidelines. All experimental procedures were approved by the Committee for Ethics in Research on Human Beings of the Bauru School of Dentistry, University of São Paulo (number 001/2012).

### 2.2. Isolation and Co-Culture of Human Palate Epithelial Cells

Volunteers were considered for inclusion in our study if they presented with normal health and did not suffer from any of the following conditions: diabetes mellitus, alcoholism, tobacco usage, periodontal disease or other oral pathology, gingival bleeding, and immune/endocrine/hematological alterations and the use of xerostomia, antifungal, or antibiotic medications, respectively. After disinfection of the samples in 70% alcoholic solution and the mechanical separation of epithelium from connective tissue, HPECs were obtained using either the direct explant method or the enzymatic method [26].

#### 2.2.1. Direct Explant Method

The separate samples for the explant method were cut into small fragments (1 mm^2^) and placed in culture dishes with the epithelial surface facing the plate. Then, the fragments were slightly humidified in a culture medium of Dulbecco’s Modified Eagle Medium (DMEM; Gibco^®^ Invitrogen, Grand Island, NY, USA) supplemented with 10% fetal bovine serum (FBS; Gibco^®^ Invitrogen, Grand Island, NY, USA) and penicillin/streptomycin (100 IU/mL/100 μg/mL; Gibco^®^ Invitrogen, Grand Island, NY, USA), which was dropped between the fragments. The plates were incubated in a humidified atmosphere at 37 °C with 5% CO_2_ for approximately 24 h. After this period, the fragments were flooded with an appropriate keratinocyte culture medium constituted of DMEM and F12 Nutrient Mixture (Ham; Gibco^®^ Invitrogen, Grand Island, NY, USA) in a 2:1 ratio, with 10% Bovine Serum Product Fetal Clone III (Hyclone, Logan, UT, USA), penicillin/streptomycin (100 IU/mL/100 μg/mL), glutamine (4 mM; Gibco^®^ Invitrogen, Grand Island, NY, USA), adenine (0.18 mM; Sigma Chemical Co., St. Louis, MO, USA), insulin (5 μg/mL; Sigma Chemical Co., St. Louis, MO, USA), hydrocortisone (0.4 μg/mL; Sigma Chemical Co., St. Louis, MO, USA), cholera toxin (0.1 nM; Sigma Chemical Co., St. Louis, MO, USA), and triiodothyronine (20 pM; Sigma Chemical Co., St. Louis, MO, USA). The culture plates were examined daily using an inverted microscope. When the migrated cells reached a diameter of 2–5 mm, Epidermal Growth Factor (EGF, 10 ng/mL; R&D Systems, Minneapolis, MN, USA) was also added to the DMEM/HamF12 medium. When the cells around the sample fragment started to change their morphology and increase their volume, they were removed by trypsinization (0.05% trypsin/0.02% EDTA; Gibco^®^ Invitrogen, Grand Island, NY, USA) and their further propagation in culture was started.

#### 2.2.2. Enzymatic Method

The initial fragments were cut into smaller pieces (0.5 × 0.5 mm) and immersed in a 0.05% trypsin/0.02% EDTA solution under low agitation at 37 °C. At intervals of 30 min, the supernatant cells were collected, the trypsin was neutralized with DMEM medium containing 10% FBS and penicillin/streptomycin (100 UI/mL/100 μg/mL), and this mixture was centrifuged at 1500 rpm for 5 min. The cells were resuspended in a keratinocyte culture medium (DMEM/HamF12, 2:1) supplemented as described in Section 2.2.1. The cells (6 × 10^3^ cells/cm^2^) were seeded on a mitotically inactivated human gingival fibroblasts feeder-layer. The integrated keratinocytes/feeder-layer culture was kept in a humid incubator (5% CO_2_/95% air, 37 °C) with a DMEM/HamF12 culture medium supplement. EGF (10 ng/mL) was added to the DMEM/HamF12 culture medium after it was first changed.

#### 2.2.3. Mitomycin C Treatment of Human Fibroblasts

Human gingival fibroblasts were isolated from connective tissue, mechanically separated from the donated epithelial fragments, and cultured in DMEM culture medium supplemented with 10% FBS and penicillin/streptomycin (100 IU/mL/100 μg/mL). Fibroblasts used as feeder-layers (1.5 × 10^4^ cells/cm^2^) were mitotically inactivated with mitomycin C (8 μg/mL; Sigma Chemical Co., St. Louis, MO, USA) diluted in 10% SBF DMEM growth medium after incubation for 2 h at 37 °C. Then, the cells were washed, resuspended in culture medium, and seeded into cell culture bottles [27].

#### 2.2.4. Propagation of Keratinocytes on the Feeder-Layer

After a keratinocyte primary culture was developed using both methods, the propagation of these cells in subsequent cultures was carried out. The passages were fulfilled each time the culture reached the subconfluence stage. At that moment, trypsinization and centrifugation were performed. The integrated keratinocytes/feeder-layer culture was first trypsinized for 5 min to remove the fibroblasts that had been discarded. The keratinocytes were then removed by trypsinization for 15 min, counted, and seeded (6 × 10^3^ cells/cm^2^) on a new feeder-layer. The medium was changed every 2–3 days [26].

### 2.3. OBA-9 Cell Culture

Cells from the immortalized human gingival epithelial cell line OBA-9 were obtained from the Institute of Biomedical Sciences (ICB-USP). The cells were subcultured in T25 Cell coat (Greiner Bio-One, Americana, SP, Brazil) containing keratinocyte culture medium (DKSFM, ThermoFisher, Waltham, MA, USA) supplemented with penicillin/streptomycin (100 IU/mL/100 μg/mL) and were kept in a humid incubator (5% CO_2_/95% air, 37 °C). Cells were trypsinized with Tryple Express (Thermo Fisher), plated at 10^4^ cell/well in 96-well plates for the quantitative lactate dehydrogenase (LDH) assay, and plated at 10^5^ cells/well in 24-well plates for the release of reactive oxygen species assay.

### 2.4. Fungal Growth Conditions and the Generation of Supernatants from C. albicans

*C. albicans* ATCC 90,028 was used for this study. Yeast cells were grown in trypticase soy broth medium (TSB, Himedia, Mumbai, Maharashtra, India) supplemented with 0.5% chloramphenicol (0.05 mg/mL; Sigma Chemical Co., St. Louis, MO, USA) for 24 h at 37 °C under gentle agitation. Then, the yeast cells were washed with phosphate-buffered saline (PBS), counted using a hemocytometer, resuspended in the appropriate keratinocyte culture medium (DMEM/HamF12, 2:1), and supplemented as described in Section 2.2.1, but without an antibiotic. Concentrations of 10^3^, 2.5 × 10^3^, 4 × 10^3^, 10^4^, and 4 × 10^4^ yeast cells/mL were used to test HPEC (10^5^ cells/mL) interaction with *C. albicans* in the fungal proportions 1/100, 1/40, 1/25, 1/10, and 1/2.5 *Candida*/HPEC, respectively [28]. The fungal proportions 1/100, 1/40, and 1/10 *Candida*/cells were used to challenge cells of the OBA-9 cell line.

To analyze the effects of supernatant from *C. albicans* on HPECs, yeast cells were cultured with DMEM/HamF12 (2:1) medium supplemented as described in Section 2.2.1 but without antibiotics, to obtain the hyphal form, for 24 h at 37 °C in the different *Candida*/HPEC ratios. These conditioned media were obtained by centrifugation (10,000 rpm for 10 min) and used in additional experiments [28]. We did not evaluate the effects of hyphae supernatant on the OBA-9 cells.

### 2.5. HPEC Interaction with C. albicans via Either Direct (D.C.) or Indirect (I.C.) Cell–Cell Contact by Means of the Fungal Supernatants

For the experiments, 10^5^ HPEC cells/mL were seeded on culture plates and incubated (5% CO_2_/95% air, 37 °C) in complete DMEM/HamF12 (2:1) medium supplemented as described in Section 2.2.1 for 24 h. After this period, the keratinocyte culture medium was discarded, and the cells were either kept together with *Candida* supernatants, as a form of indirect contact (IC), or in direct contact with *C. albicans* cells (DC) for an intercalated experimental time starting from 3 h to a maximum of 24 h. HPECs maintained in DMEM/HamF12 (2:1) medium supplemented as described in Section 2.2.1 but without an antibiotic were used as an experimental control (CRTL/Medium, negative control). For the DC experiment, *Candida* cell cultures were added to HPECs [28,29]. For the IC experiment, keratinocytes were incubated with the hyphae supernatant that was obtained through the fungal culture [28].

### 2.6. HPEC Viability Assays

To evaluate the effect of *C. albicans* on HPEC viability, a LIVE/DEAD cell viability assay was performed (Invitrogen, Carlsbad, CA, USA) according to the manufacturer’s instructions. HPECs were seeded onto a six-well culture plate and stimulated directly (DC) or indirectly (IC) with *C. albicans* (1/100, 1/40, 1/25, 1/10, and 1/2.5 *Candida*/HPEC), under the conditions described in Section 2.5, for 3, 6, 8, 10, 12, and 24 h. After the epithelial cells were stained (1 μL/mL of dye in PBS) for 20 min at 37 °C under light protection, a quantitative analysis was performed using an inverted fluorescence microscope (Leica DM IRBE, Mannheim, Baden-Württemberg, Germany) through 10 randomly captured fields per well at 200× magnification. The number of living cells expressed as a percentage of the total cell number was used to establish the quantitative cell viability index.

### 2.7. HPEC Invasion by *C. albicans* Post Direct Contact

HPECs were kept in DC with *Candida* fungus (1/100, 1/40, 1/25, 1/10, and 1/2.5 *Candida*/HPEC) in 24-well plates containing sterile spherical glass coverslips (13 mm in diameter) for 3, 6, 8, 10, 12, and 24 h. The wells were washed with PBS to remove the extracellular fungus. The coverslips to which cells were attached were fixed with 2% paraformaldehyde in PBS for 15 min and washed again with PBS. Then, they were stained with 0.05 mg/mL acridine orange (Merck, Darmstadt, Germany) for 15 min in a dark room. After this, the coverslips were carefully removed and placed on glass slides with mounting medium. The slides were qualitatively evaluated by confocal laser-scanning microscopy (TCS-SPE, Leica, Wetzlar, Germany) at 630× magnification, which allowed us to differentiate the fungus inside the keratinocytes from those that were only bounded to the HPEC surface [30]. The experiments were also performed in the absence of epithelial cells to compare the fungal biofilm that formed with those that developed in the presence of HPECs.

### 2.8. HPEC Apoptosis Assay

The cells to be analyzed for apoptosis were stained with Hoechst 33,258 [31]. Initially, HPECs were kept in DC or IC in 24-well plates at 1/100, 1/40, and 1/10 *Candida*/HPEC for 3, 6, and 10 h. After washing, cells were incubated with Hoechst 33,258 (10 μg/mL in PBS; Invitrogen, Carlsbad, CA, USA) for 20 min at 37 °C to stain the DNA in each cell. Then, individual nuclei were visualized at 400× magnification to distinguish the normal uniform nuclear pattern from the characteristic condensed coalesced chromatin pattern of apoptotic cells [20]. For this, 10 random microscopic fields per well were analyzed in an inverted fluorescence microscope. HPEC apoptosis is expressed as a percentage of overall cell numbers.

### 2.9. OBA-9 Cell Line D.C. Interaction and Lactate Dehydrogenase (LDH) Release Assay 

To verify our previously obtained results, which revealed that the *C. albicans* fungus has a slight cytotoxic effect on epithelial cells in vitro, a highly sensitive method that can measure low numbers of cells undergoing apoptosis [32] was used to analyze OBA-9 cells. For this purpose, DC-induced cytotoxicity was measured using the CytoTox 96 Non-Radioactive Cytotoxicity Assay (Promega Corporation, Madison, WI, USA). The standard protocol for the assays reported here was performed according to the manufacturer’s instructions. This colorimetric assay was used to quantitatively measure the LDH that was released into the media from damaged cells as a biomarker of cellular cytotoxicity and cytolysis. OBA-9 cells were plated in a 96-well plate (10^4^ cells per well) in culture medium supplemented with 10% fetal bovine serum and incubated overnight at 37 °C in 5% CO_2_. The cells were then challenged with different concentrations of *Candida* (1/100, 1/40, and 1/10 *Candida*/OBA) for 3, 6, and 10 h before being subjected to the indicated assays. OBA-9 cells maintained in DKSFM medium supplemented as described in Section 2.2.1 but without an antibiotic were used as the negative control (CRTL/Medium). The kit provided the LDH positive control. LDH activity was determined by spectrophotometric absorbance using a standard plate reader with a reference wavelength of 490 nm.

### 2.10. NO Production by HPEC and OBA-9 Cells

For NO detection in the experimental samples, nitrite (NO^−^_2_) production was measured in the supernatants using the Griess method [33]. The DC and IC assays were performed at ratios of 1/100, 1/40, and 1/10 *Candida*/HPEC for 3, 6, and 10 h. Briefly, 100 μL of supernatant sample was incubated with an equal volume of the Griess reagent (Sigma Chemical Co., St. Louis, MO, USA) at room temperature for 30 min. The absorbance was measured using a microplate reader (Spectra Max 250, Molecular Devices, Sunnywale, CA, USA) at 540 nm. The NO^-^_2_ concentration was determined using a standard curve for NaNO_2_ at a concentration range from 0.2 to 1.5 μM. The NO^-^_2_ production by OBA-9 cells in the DC assay was also determined using the Griess method but using lipopolysaccharide (LPS) endotoxin as a positive control (CTRL/LPS).

### 2.11. Reactive Oxygen Species (ROS) Production by OBA-9 Cells

The amount of endogenous ROS in OBA-9 cells was determined using the fluorescent probe 2’,7-dichlorofluorescin diacetate (Cell Rox Deep Red Reagent; Life Technologies, Grand Island, NY, USA). OAB-9 cells (10^5^ cells/well in a 24-well plate) were DC incubated with *C. albicans* (1/100, 1/40, and 1/10 *Candida*/OBA) for 3, 6, and 10 h (5% CO_2_/95% air, 37 °C). Cells in medium and Phorbol 12-myristate 13-acetate (PMA) were the negative (CTRL/Medium) and positive (CTRL/PMA) controls, respectively. Afterward, they were incubated with 5 μM of Cell Rox (5% CO_2_/95% air, 37 °C) for 30 min. The fluorescence intensities (FIs) of the cells were measured using the multiplate reader at 640/665 nm at 37 °C.

### 2.12. HPEC hBD-2 Gene Expression (RT-qPCR)

For the *hBD-2* gene expression evaluation, HPECs were seeded in 96-well plates at a density of 10^4^ cells per well. The DC and IC experiments were performed at 1/100, 1/40, and 1/10 *Candida*/HPEC for 3, 6, and 10 h. LPS from *Escherichia coli* (1 μg/mL) was used as a control (CTRL/LPS) under the same conditions. After the experimental period, RNA was extracted for reverse transcription (RT) followed by a quantitative polymerase chain reaction (RT-qPCR) analysis. Total RNA and complementary DNA (cDNA) were directly obtained from epithelial cells using a Cells-to-CT^TM^ Kit (Ambion Inc., Life Technologies, Austin, TX, USA) according to the manufacturer’s instructions. In brief, the cells were washed two times with 1× PBS and incubated for 5 min with the 49.5 μL of lysis solution and 0.5 μL of DNAse that are provided with the kit. Following this incubation, 5 μL of stop solution was added for 2 min, and the RT was performed by adding the 2× RT buffer and the 20× enzyme mix to the lysate followed by incubation at 37 °C for 60 min and a subsequent 95 °C step for 5 min to stop the reaction. The qPCR was performed using TaqMan Gene Expression PCR Master Mix (Applied Biosystems, Life Technologies, Warrington, UK) and a proprietary primer (Applied Biosystems, Life Technologies, Warrington, UK) targeting mRNA of hBD-2 (Hs00175474_m1, forward 5’ CCAGCCATCAGCCATGAGGGT 3’ and reverse 5’ GGAGCCCTTTCTGAATCCGCA 3’). All experiments were performed in an aViiA 7 Real-Time PCR system (Applied Biosystems, Life Technologies, Warrington, UK) using the comparative cycle threshold (Ct) method (ΔΔCt). The human β-globulin gene was used as the reference gene for all of the reactions because it was the most stable reference gene in our experiments compared with β-actin (data not shown).

### 2.13. HPEC hBD-2 Production (ELISA)

The hBD-2 concentration was determined in the cell-free supernatants that were obtained after 3, 6, and 10 h of HPEC culture in DC and IC with *C. albicans* at 1/100, 1/40, and 1/10 *Candida*/HPEC. The utilized method was an enzyme-linked immunosorbent assay (ELISA) using a Human BD-2 ELISA Construction Kit (Antigenix America, Melville, NY, USA). The evaluations were performed according to the manufacturer’s instructions. The absorbance was read at 450 nm using a multiwell scanning spectrophotometer (ELISA reader; Amersham, Pharmacia Biotech, Cambridge, UK).

## 3. Results

### 3.1. Characterization of Candida/HPEC Proportion and Experimental Time by Cell Viability

**Figure 1:**
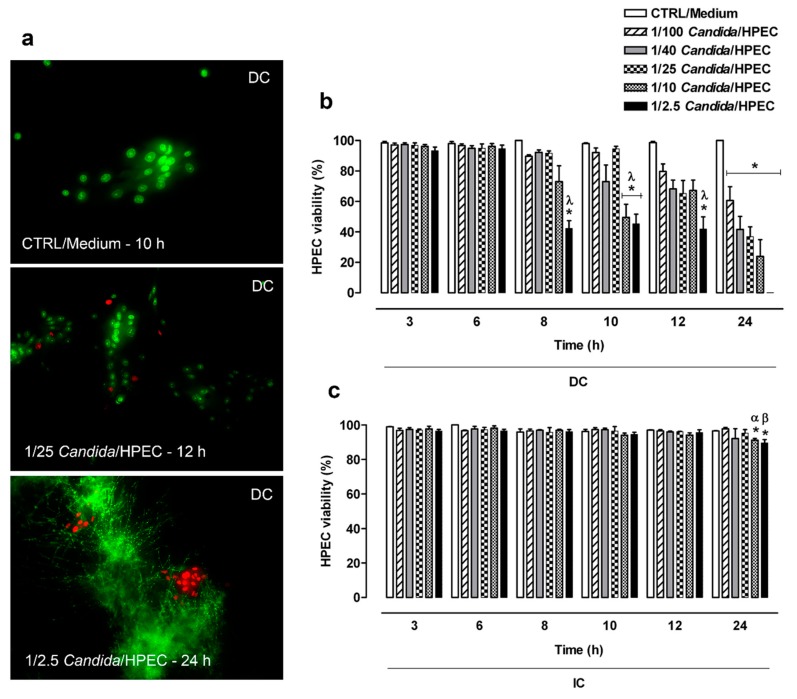
Human palate epithelial cell (HPEC) viability after direct contact (DC) with different *Candida albicans*/HPEC ratios (1/100 to 1/2.5) over time (3–24 h). (**a**) Representative fluorescence images of viable HPECs stained with calcein (green fluorescence, living cells) after direct contact with *Candida* cells and non-viable HPECs with ethidium bromide linked to DNA (red fluorescence nuclei, dead cells). * Fungus in filamentous form. HPECs cultured in medium (cells unchallenged) served as a control (CTRL/Medium). LIVE/DEAD staining (fluorescence microscope, ×200 magnification). (**b**,**c**) The number of living cells expressed as percentage of the total cell number was used to establish the quantitative cell viability index. Comparative analysis among different fungal ratios after DC or indirect contact (IC) over time. (*) *p* < 0.05 compared to the medium at the same time. (λ) *p* < 0.016 compared to 3 and 6 h. (α) *p* < 0.03 compared to 3, 6, and 8 h. (β) *p* < 0.05 compared to all other times. Results are expressed as the mean ± standard error of the mean (SEM) of at least three independent experiments performed in triplicate with the factorial ANOVA contrast test.

In Figure 1a, viable HPECs are highlighted by calcein absorption and dead cells by ethidium bromide linked to DNA, resulting in a cell viability index. Regarding the DC assay (Figure 1b), the cell viability assay showed that the HPECs maintained viability for over 12 h when challenged with the lowest fungal proportions (1/100, 1/40, and 1/25 *Candida*/HPEC). Accordingly, an increase in the fungus concentration resulted in HPEC death from 8 h of culture onward. The highest proportions (1/10 and 1/2.5 *Candida*/HPEC) caused significant epithelial cell death from 10 h onward compared to the negative control, CTRL/Medium, and early periods of culture (3 and 6 h). At 24 h, all evaluated fungal ratios resulted in significant epithelial cell death in comparison to the control medium as shown in Figure 1b.

HPEC viability in the IC assay (Figure 1c) was maintained for over 12 h under all experimental *Candida*/HPEC proportions. Unlike the DC assay, the IC assay showed only the highest ratios (1/10 and 1/2.5 *Candida*/HPEC) at 24 h, resulting in significant HPEC death compared to the CTRL/Medium and earlier periods (Figure 1c). Briefly, both the DC and IC assays resulted in HPEC death mainly in later periods and under greater *Candida*/HPEC proportions. On the other hand, the cell viability in the DC assay was significantly lower than that in the IC assay under all fungal proportions at 24 h.

### 3.2. HPEC Invasion by C. albicans after Direct Contact

**Figure 2:**
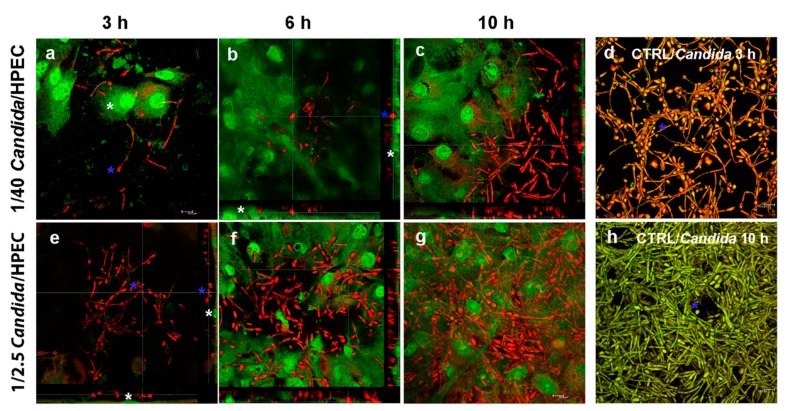
The *C. albicans* - human palate epithelial cell (HPEC) invasion process after direct contact (DC) with different ratios of *Candida*/HPEC. (**a**,**e**) Filamentous fungus (blue asterisk) at the periphery of epithelial colonies; filaments positioned on the epithelial cells (white asterisk) without epithelial invasion. (**b**) Initial intraepithelial invasion (active epithelial penetration) by the fungus (blue asterisk). HPECs are stained in green (white asterisk). (**c**,**f**,**g**) Intensified intraepithelial invasion due to the increased *C. albicans* concentration and density of prolific filamentous forms over time. (**d**,**h**) The fungal biofilm (blue asterisk) in the absence of epithelial cells (CTRL/*Candida*) that is qualitatively more intense than the one in the presence of HPECs. Acridine orange staining (confocal laser-scanning microscopy, 630× magnification).

A qualitative evaluation showed that the *C. albicans* HPEC invasion process progressed through three different stages. Initially, at 3 h, numerous filamentous fungi (red) were observed in the peripheral region of the epithelial colonies (green) (Figure 2a). The number of filaments increased proportionally to the *Candida*/HPEC proportion. Although some fields showed filaments that were positioned on the epithelial cells, no epithelial invasion was observed until 3 h of culture (Figure 2e). After 6 h, an initial intraepithelial invasion (active epithelial penetration) by the fungus was observed from the 1/40 *Candida*/HPEC (Figure 2b). Epithelial penetration was enhanced with increasing *C. albicans* concentration (Figure 2f), and a separate evaluation of each proportion showed that this invasion was amplified over time (Figure 2c). Therefore, once the initial fungus–epithelial cell contact was established, the degree of active epithelial penetration increased simultaneously with the numeric increase in prolific filamentous forms (6–12 h). Significant epithelial cell damage was observed after 10 h, which was characterized by intracellular filamentous fungi and epithelial cell disintegration. These characteristics were aggravated after contact with the highest fungal proportions (1/10 and 1/2.5 *Candida*/HPEC) for the remainder of the experimental time (Figure 2g). It is worth noting that no intraepithelial invasion occurred under the lowest proportion (1/100 *Candida*/HPEC), regardless of the contact time. In the absence of epithelial cells, the fungal biofilm was shown to be qualitatively more intense than that grown in the presence of HPEC, regardless of the tested proportion or time (Figure 2d,h).

### 3.3. HPEC Apoptosis by *C. albicans*

**Figure 3:**
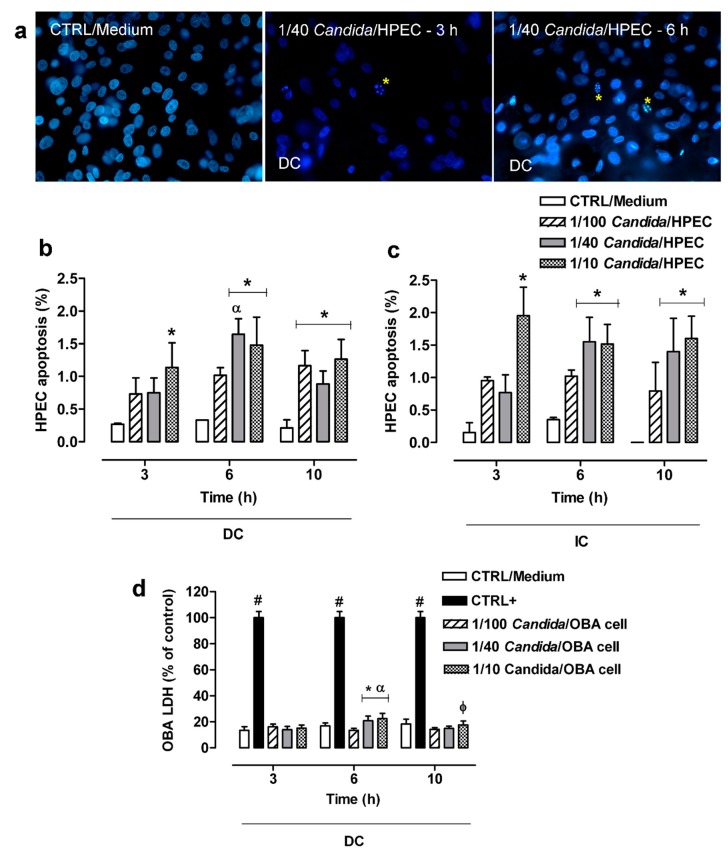
Cytotoxic effects induced in human palate epithelial cells (HPECs) by different *C. albicans* ratios over time. (**a**) HPECs underwent typical morphological apoptosis after direct contact (DC) with *C. albicans*. The DNA-binding dye showed nuclear chromatin morphological features as a quantitative apoptosis index within the cell culture system. * Apoptotic bodies. Hoechst 33,258 staining (fluorescence microscope, ×400 magnification). (**b**,**c**) HPEC apoptosis analysis between different fungal ratios after DC or indirect contact (IC) with *C. albicans* over time. (**d**) Cytotoxic effects were measured by lactate dehydrogenase (LDH) enzymatic activity in cells of the immortalized human gingival epithelial cell line OBA-9 after DC with the fungus (*) *p <* 0.02 compared to unchallenged cells (CTRL/Medium) at the same time. (#) *p* < 0.001 compared to all *Candida*/OBA cell ratios at the same time. (α) *p* < 0.04 compared to all other times. (φ) *p* < 0.03 compared to 1/100 *Candida*/OBA cells at 10 h. Results are expressed as the mean ± SEM of at least three independent experiments performed in triplicate with the factorial ANOVA contrast test.

HPEC apoptosis was determined using a DNA-binding dye that characterized the nuclear chromatin morphological features that are compatible with cell apoptosis, resulting in the index within the cell culture system shown in Figure 3a. HPECs showed a low (less than 2%) apoptosis percentage at all fungal concentrations (1/100, 1/40, and 1/10 *Candida*/HPEC) over 10 h of incubation. Despite the low apoptosis percentage, DC with 1/40 *Candida*/HPEC at 6 h resulted in cell apoptosis percentage values that were significantly higher than those obtained under the same experimental conditions at 3 h (Figure 3b). The HPEC apoptosis process began earlier with increasing fungus concentration. Thus, the 1/10 *Candida*/HPECs at 3 h showed significant HPEC apoptosis compared to the CTRL/Medium. Moreover, all tested fungal proportions induced significant HPEC apoptosis at 10 h (Figure 3b).

Unlike the DC assay, in which an apoptosis peak was observed after 6 h, the IC assay showed similar HPEC apoptosis percentage values for each fungal proportion analyzed separately over time (Figure 3c). On the other hand, similar to the DC assay, the HPECs in IC with *C. albicans* cells under the highest fungal proportions (1/40 and 1/10 *Candida*/HPEC) showed a significant increase in the number of apoptotic cells compared to the CTRL/Medium at 6 and 10 h (Figure 3c). No significant difference was found between DC and IC in the apoptosis assay when each proportion was individually evaluated at the same time.

### 3.4. LDH Release by OBA-9

Figure 3d shows the results of the LDH cytotoxicity assay. The DC cytotoxicity effect was low (around 20%) at all *Candida*/OBA ratios and differed significantly from the positive control. Despite the low cytotoxicity, higher LDH release was detected in the 1/40 and 1/10 *Candida*/OBA cells at 6 h, with a significant increase in cytotoxicity compared to the CTRL/Medium (negative control) and the 1/100 *Candida*/OBA cells. This LDH release peak after 6 h of incubation differed significantly from those at 3 and 10 h of incubation. After 10 h of incubation (Figure 3d), the 1/10 *Candida*/OBA cells had higher cytotoxicity than the 1/100 *Candida*/OBA cells.

### 3.5. NO Production by HPEC and OBA-9 Cells

**Figure 4:**
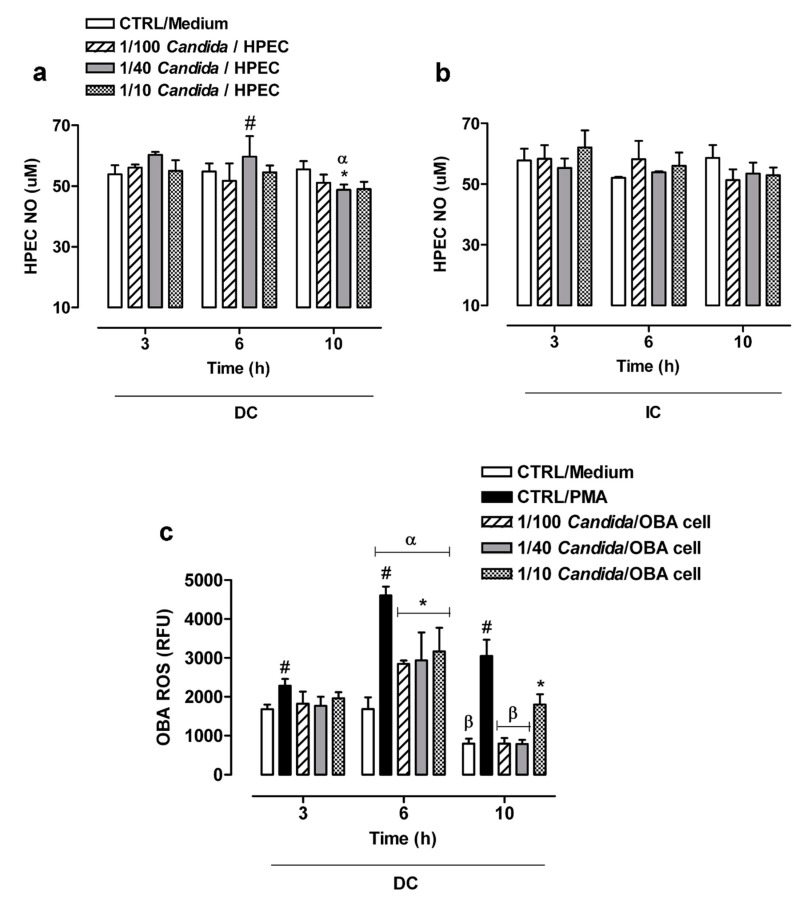
Nitric oxide (NO) and reactive oxygen species (ROS) production by cells treated with different *C. albicans* ratios over time. (**a**,**b**) Comparative analysis of the NO production by human palate epithelial cells (HPECs) among different fungal ratios after direct (DC) or indirect contact (IC) with the fungus. (**c**) Comparative analysis of the ROS production by immortalized human gingival epithelial cell line OBA-9 among different fungal ratios after DC with the fungus. (*) *p* < 0.05 compared to unchallenged cells (CTRL/Medium) at the same time. (#) *p* < 0.05 compared to all other *Candida*/cell ratios and the CTRL/Medium at the same time. (α) *p* < 0.009 compared to all other times. (β) *p* < 0.001 compared to 3 h of incubation. Results are expressed as the mean ± SEM of at least three independent experiments performed in triplicate with the factorial ANOVA contrast test.

The NO production by HPECs in DC with *C. albicans* was found to be similar to the basal concentrations (CTRL/Medium) over time, except for the 1/40 *Candida*/HPEC proportion. It is worth noting that the 1/40 *Candida*/HPECs at 6 h showed a significant increase in NO values compared to the CTRL/Medium and other fungal proportions (Figure 4a). However, this NO level was not maintained over time; the 1/40 *Candida*/HPECs at 10 h showed a significant reduction in the substrate compared to the CTRL/Medium and other time periods (Figure 4a).

The NO production by HPECs in IC with the fungus remained at similar levels from 3 to 10 h for all *Candida*/HPEC proportions. An increase in the fungus concentration did not change the HPEC NO production profile, which was similar to that of the CTRL/Medium (Figure 4b). The NO production was found to be similar between DC and IC when considering the same time period and each individual fungal proportion. Unlike the HPEC NO production, the OBA-9 cells did not show either a basal NO concentration or NO production after challenge with the fungus.

### 3.6. ROS Production by OBA-9 Cells

Considering the initially observed slight variation in or absence of NO production by the epithelial cells when challenged with *C. albicans*, ROS production was subsequently evaluated in OBA-9 cells (Figure 4c). ROS production by these cells in DC with *C. albicans* was similar to the basal concentration (CTRL/Medium) at 3 h for all analyzed *Candida*/OBA ratios; only the CTRL/PMA induced significant ROS production compared to untreated cells. Activation of OBA-9 cells by *C. albicans* at 6 h resulted in a significant ROS release peak as compared to the basal concentration over all tested fungal proportions; however, it was lower than that in the CTRL/PMA. This ROS release peak after 6 h of incubation differed significantly from those after 3 and 10 h of incubation. After 10 h of incubation, the 1/100 and 1/40 *Candida*/OBA cells were found to have reduced ROS production levels that had returned to baseline levels and were statistically significantly lower than the ROS production levels at 3 h of incubation. However, the 1/10 *Candida*/OBA cells at 10 h had higher ROS production levels than those of the other fungal proportions, below only the ROS production levels of cells under PMA stimulation (Figure 4c).

### 3.7. HPEC hBD-2 Gene Expression (RT-qPCR)

**Figure 5:**
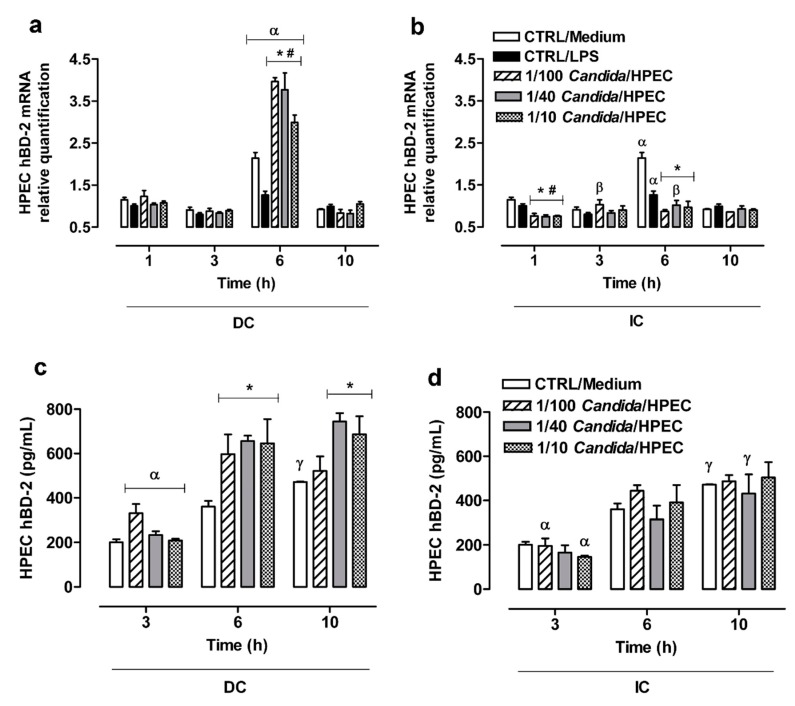
Significant β-defensin 2 (*hBD-2*) expression by human palate epithelial cells (HPECs) was induced by *C. albicans* infection. (**a**,**b**) Regulation of *hBD-2* mRNA expression in HPECs after direct (DC) or indirect contact (IC) with *C. albicans* in different ratios over time. The mRNA levels were quantified by RT-qPCR using gene expression assays and normalized with β-globulin as the reference gene. (**c**,**d**) hBD-2 secretion by HPECs after DC and IC with *C. albicans* over time. (*) *p* < 0.007 compared to unchallenged cells (CTRL/Medium) at the same time. (#) *p* < 0.047 compared to CTRL/LPS at the same time. (α) *p* < 0.026 compared to all other times. (β) *p* < 0.001 compared to 1 h. (γ) *p* < 0.005 compared to 3 h. Results are expressed as the mean ± SEM of at least three independent experiments performed in triplicate with the factorial ANOVA contrast test.

Since HPECs may show a gene expression peak after a short period of contact with a fungus, *hBD-2* gene expression by these cells against *C. albicans* was also evaluated at 1 h in addition to other selected experimental times. Maximum expression was found to occur at 6 h of incubation in the DC assay or in the presence of LPS or only of the culture medium (Figure 5a). The values were statistically different to those obtained at other time periods. At 6 h of incubation, all *Candida*/HPEC ratios were found to have induced a significant increase in *hBD-2* expression compared to the baseline CTRL/Medium (Figure 5a).

A higher *hBD-2* gene expression was noted in the IC assay with the 1/40 *Candida*/HPEC ratio at 6 h, as well as the 1/100 *Candida*/HPEC ratio at 3 h compared to the initial time at 1 h (Figure 5b). In contrast to DC, IC induced a lower molecular expression compared to the baseline at 1 and 6 h for all tested fungal proportions. The contact with soluble *Candida* factors resulted in *hBD-2* expression that was similar to basal production at 3 and 10 h (Figure 5b). A comparison of the DC and IC assays shows that DC induced significantly (*p* < 0.05) higher *hBD-2* expression at 6 h compared to IC for all analyzed fungal proportions. After 1 h, the *hBD-2* gene expression in the DC assay was significantly (*p* < 0.05) higher than that in the IC assay for the 1/40 and 1/10 *Candida*/HPEC proportions.

### 3.8. hBD-2 Production by HPECs

The hBD-2 production by HPECs at 3 h under DC with *C. albicans* was found to be similar to the baseline hBD-2 production level (CTRL/Medium) for all analyzed *Candida*/HPEC ratios (Figure 5c). However, after 6 h of incubation, all tested fungal ratios were found to induce a significant hBD-2 production peak compared to unchallenged cells. The cells in the highest fungal proportions (1/40 and 1/10 *Candida*/HPEC) maintained this hBD-2 production over 10 h of incubation. This increase in hBD-2 production at 6 and 10 h differed from the hBD-2 production at the initial time of 3 h, regardless of the *Candida*/HPEC ratio (Figure 5c).

In contrast to DC, IC did not induce a significant hBD-2 production level as compared to unchallenged HPECs for all analyzed time periods. However, the results showed an increase in hBD-2 production over time that kept up with the baseline level, which differed from the level at the initial time of 3 h (Figure 5d). A comparison of the DC and IC assays shows that, in the DC assay, the highest fungal proportions (1/40 and 1/10 *Candida*/HPEC) induced significantly (*p* < 0.05) higher hBD-2 production at 6 and 10 h than in the IC assay.

## 4. Discussion

In this study, we reported the survivability and immune responses of HPECs against in vitro direct contact with *C. albicans* cells (DC assay) or hyphae supernatant from *C. albicans* (IC assay) over time. Infected HPECs showed significant hBD-2 peptide production, which is an important antimicrobial defense mechanism, as well as an increase in NO production in one of the evaluated time periods. On the other hand, HPECs showed a decrease in cell viability at higher *Candida*/HPEC proportions, as well as under conditions of prolonged contact with the fungus or its hyphae supernatant. Furthermore, the contact between epithelial cells and fungal cells allowed the fungal cells to transition to their filamentous pathogenic form, proliferate, and penetrate into the epithelium. Moreover, the HPECs showed slight apoptosis and reduced NO production over time, particularly in the 1/40 *Candida*/HPEC ratio. As expected, the observed changes occurred mainly after the DC assay.

Although many studies that have evaluated the effect of *C. albicans* on oral epithelium can be found in the literature [10,19,20,21,22,23], no previous study, to our knowledge, involved a HPECs primary culture. This seems to be the first report on the influence of *C. albicans* on the epithelial biological response of HPECs primary. In most cases, the polymerized resin of maxillary dentures is contaminated by the *Candida* fungus, resulting in an inflammatory reaction of the palatal mucosa known as DS, prosthetic stomatitis, or *Candida*-related DS [1,2]. Our infection model was able to mimic the palatal epithelium response in maxillary denture wearers. It should be possible to extend the current studies to obtain more comprehensive results on *C. albicans* pathogenicity mechanisms and host immune factors for the control of DS development.

Studies on *Candida*-related DS have used epithelial cell lines derived from well-differentiated oral carcinomas (SCC15, ATCC; TR146; FaDu) [10,19,20,21] and immortalized human oral keratinocyte cell lines (OKF6/TERT2) created from telomerase 2 forced expression [22,23]. Other studies of human oral diseases have been performed with normal oral epithelial cells immortalized by transduction with simian virus 40 (SV40), such as OBA-9 cells [34,35,36], which were used in this study. However, the use of SV40 results in effects that disrupt DNA repair in virally immortalized cell lines [35,36], affect apoptosis signaling, and induce the accumulation of mutations in some cells. This implies that they have additional effects other than immortalization [36,37], and highlights the importance of the results obtained in this study with an HPEC primary cell culture.

Firstly, in this work, preliminary HPEC viability and penetration tests after contact with *C. albicans* allowed for the selection of *Candida*/HPEC proportions and experimental times for the apoptosis, NO production, and hBD-2 production analyses. The results led to the selection of proportions of 1/100, 1/40, and 1/10 *Candida*/HPEC, and 3, 6, and 10 h experimental periods, to ensure that cells remained viable for further analysis. Direct interaction between *C. albicans* cells HPECs occurred in phases of adhesion, invasion, and cell damage, similar to other studies [8,9]. On the other hand, in spite of the increase in cell damage with epithelial penetration by the fungal cells in their filamentous form, the qualitative assessment of the HPECs suggested that an antimicrobial defense response occurred against the fungus. Initially, the fungal filaments were observed only on the periphery of the epithelial colonies, indicating an initial colonization phase with fungal cell adhesion to the host cell, but suggesting that the HPECs repulsed the fungus. Also, in the absence of HPECs (CTRL/*Candida* for 3 h of incubation), the fungal biofilm was observed to have a more intense presence and occupy the entire area of the culture plate, which was not observed in the biofilm developed in the presence of epithelial cells. In agreement with this finding, researchers have reported that epithelial cell lines are able to control fungal cell growth and invasion of tissue [8] through their fungistatic activity [14], and are even able to stop the progression of apoptosis [22]. An intraepithelial invasion phase (active epithelial penetration) associated with the yeast-to-fungal filamentous transition was observed at 6 h, in particular in the 1/40 *Candida*/HPEC proportion, which intensified over time as the number of prolific filamentous forms increased. Finally, in a later phase (10 h), the fungus in its higher proportions resulted in significant epithelial cell death, coinciding with intracellular filamentous fungi and epithelial disintegration, and similar to an in vitro model using the oral epithelia TR146 line [10,38].

Therefore, in our experimental model, contact between *C. albicans* and HPECs in the ratio 1/40 *Candida*/HPECs for 6 h caught our attention. Under these conditions, we observed an initial active epithelial penetration by the fungus simultaneous with significant epithelial apoptosis. However, although the epithelial cell death percentage did not exceed 2%, the apoptotic test demonstrates that, at an early stage, keratinocytes from the primary palate culture suffered from *C. albicans* invasion. Studies have shown that apoptosis of oral epithelial cells by *C. albicans* depends on its ability to physically interact with and invade the host cell [19,20]. On the other hand, the results of studies with keratinocytes from human skin (the HaCaT line) [24] suggest that apoptosis induction depends on factors that the viable pathogen releases. Both DC and IC resulted in a similar amount of apoptosis based on our findings with primary HPECs. The morphological transformation into invasive hyphae cells is known to secrete soluble hydrolytic enzymes, such as secreted aspartyl proteinases (SAPs) [2,39] and phospholipases [24] that can promote the disruption of physical integrity followed by oral epithelial cell death [2,22,24]. Since our supernatant was obtained in the hyphae form, it is possible that it contained these soluble enzymes, which may explain the similar HPEC apoptosis observed in the DC and IC assays.

In localized oral candidiasis cases, epithelial tissue alterations occur and, consequently, host defense mechanisms activate [4]. Our group has detected high salivary NO concentrations in individuals with DS, alerting us to the importance of this radical for disease control and the prevention of *C. albicans* dissemination [40]. In response to infectious agents, keratinocyte lines release NO [6,16,41,42], suggesting active control of oral infections [11,24,43,44]. In our study, HPEC cells at a 1/40 *Candida*/HPEC ratio after 6 h of incubation in the DC assay showed a significant increase in NO release, coinciding with the initial active epithelial penetration by the fungus. However, this NO level was not maintained over time. The presence of *C. albicans* [45] and its soluble factors [24] have been shown to be capable of blocking NO production by macrophages after 24 h of incubation. In this study, it is possible that the fungus mediated the same effect on HPECs in the DC assay at later periods (10 h). Nevertheless, the IC assay did not induce NO levels above the baseline. It is possible that the aggression by the hyphae supernatant with the fungal secreted factors toward the HPECs was insufficient to stimulate a larger increase in NO levels at early periods.

To complement these analyses with primary HPECs, experiments with cell from the immortalized human gingival epithelial cell line OBA-9 were conducted, mainly for those experiments where a low or no response was detected in the primary cells. Despite the low cytotoxicity, DC induced a significant LDH release by OBA-9 cells at 6 h of incubation, similar to the HPEC apoptosis results. On the other hand, unlike the NO production by HPECs, the OBA-9 cells under DC with *C. albicans* did not produce this radical. The OBA-9 cells that were activated by *C. albicans* showed an ROS release peak at 6 h of incubation compared to basal levels. These results show that the immortalized OBA-9 cells may present distinct responses from primary culture HPECs with respect to the release of free radicals associated with the defense response.

Epithelial cells also release the antimicrobial peptides β-defensins to participate in the host defense mechanism’s activation [10,12,15] and these peptides are considered to be one of the active mediators in the control of *C. albicans* infection [13,14]. Since the hBD-2 peptide has been shown to be effective in *Candida* cell death, being the main defensin involved in the epithelium–oral environment interface [29,46], we evaluated the hBD-2 production by HPECs. The results show that the peak of *hBD-2* gene expression by HPECs occurred at 6 h of incubation under baseline conditions and was maintained after the DC assay at the ratios of 1/100 and 1/40 *Candida*/HPEC. Furthermore, this expression was found to be higher than the base level expression. Analysis of hBD-2 levels in the HPEC supernatants confirmed this observation; significantly higher hBD-2 levels were detected in all cell supernatants exposed to fungal DC at 6 h of incubation compared to CTRL/Medium. Although the *hBD-2* gene expression at 10 h returned to baseline, these data suggest an important immune defense response since the hBD-2 levels were maintained in the HPEC supernatants at the ratios of 1/40 and 1/10 *Candida*/HPEC over time. Researchers have shown an increase in *hBD-2* mRNA expression and hBD-2 secretion during double the time (12 h) of our study and concomitantly with the presence of *C. albicans* hyphae [10]; however, this expression occurred in the RHOE epithelial cell line [10], differently from our HPEC primary culture.

It is worth noting that, in the present work, this important defense response was not observed after the IC assay. This is because, after stimulation, there was a significant *hBD-2* reduction compared to basal expression, especially at 6 h. Accordingly, a significant hBD-2 level in the HPEC supernatants exposed to fungal IC was not observed compared to unchallenged cells. On the other hand, the results showed an increase in hBD-2 level over time that kept up with the baseline level. This indicates that the hyphae supernatant, which possibly contained fungal soluble secreted factors, could not increase the amount of hBD-2 expression and secretion. This implies that direct fungus–epithelium interaction is required for HPECs to activate this defense response.

Regarding the HPEC innate immune response, the experimental conditions of a 1/40 *Candida*/HPEC proportion and 6 h of incubation produced the best HPEC response, which was characterized by concomitant peaks of epithelial hBD-2 and NO production. This strengthens the previously reported idea that these in vitro conditions seem to represent the best viable fungus/cell interaction to activate immune defense mechanisms in the epithelial lining of the human palate. Thus, HPECs showed a maximal defense response against *C. albicans* that coincided with the initial active epithelial penetration by the fungus, as well as initial epithelial apoptosis signals. When the intraepithelial invasion was intensified and amplified over time by increasing the *C. albicans*/epithelium proportion, the fungus inside the epithelial cells at a later time was able to subvert cellular defense mechanisms, as highlighted by other researchers [19,21,22,47].

Although there was a good immune response after 6 h in our experimental model, the HPECs had no potential to reverse the cell invasion. This may have occurred because of the pathogenicity of the fungus for these cells, leading to a decrease in *hBD-2* expression and NO production, or due to the reduction in antimicrobial action by the HPECs, which would have allowed the proliferation of the fungus to concentrations that are lethal for epithelium. Our results with HPECs did not support the hypothesis presented by other studies that the hBD-2 antimicrobial peptide expression by the epithelium prevents *C. albicans* hyphal progression, allowing for recovery of the epithelium’s synthesis capacity and control of *Candida* spp. growth and pathogenicity [5,10].

In summary, the results of this study show that *C. albicans* can damage human palate epithelium even in the absence of direct contact between the fungus and the epithelium. On the other hand, HPECs were able to provide an antimicrobial defense response against *C. albicans* that possibly interfered with the proliferation of fungal cells. However, simultaneously to these responses, the HPECs showed the first signs of active penetration by the fungus, for which there was the need for direct fungus–epithelium interaction. Moreover, according to our findings, the activation of the palatal epithelium apoptosis pathway, induced by the fungus or its hyphae supernatant, was observed before the onset of these epithelial defense mechanisms, especially NO and hBD-2 production. It is worth highlighting the importance of this defense response by human palate epithelial cells against *C. albicans* in the first hours of contact. However, this epithelial defensive capacity was not maintained for the entire experimental period; there was discrete reduction of NO release and return of *hBD-2* gene expression to basal levels in addition to an increased epithelial invasion associated with slight apoptosis maintenance.

## References

[1] Choo K.H., Lee H.J., Knight N.J., Holmes A.R., Cannon R.D. (2017). Multilocus sequence typing (MLST) analysis of *Candida albicans* isolates colonizing acrylic dentures before and after denture replacement. Med. Mycol..

[2] Tobouti P.L., Casaroto A.R., de Almeida R.S., de Paula Ramos S., Dionísio T.J., Porto V.C., Santos C.F., Lara V.S. (2016). Expression of secreted aspartyl proteinases in an experimental model of *Candida albicans*-associated denture stomatitis. J. Prosthodont..

[3] Moosazadeh M., Akbari M., Tabrizi R., Ghorbani A., Golkari A., Banakar M., Sekhavati E., Kavari S.H., Bagheri Lankarani L. (2016). Denture stomatitis and *Candida albicans* in Iranian population: A systematic review and meta-analysis. J. Dent..

[4] Abiko Y., Saitoh M., Nishimura M., Yamazaki M., Sawamura D., Kaku T. (2007). Role of beta-defensins in oral epithelial health and disease. Med. Mol. Morphol..

[5] Weindl G., Wagener J., Schaller M. (2010). Epithelial cells and innate antifungal defense. J. Dent. Res..

[6] Gasparoto T.H., Sipert C.R., de Oliveira C.E., Porto V.C., Santos C.F., Campanelli A.P., Lara V.S. (2012). Salivary immunity in elderly individuals presented with *Candida*-related denture stomatitis. Gerodontology.

[7] Pinke K.H., Freitas P., Viera N.A., Honório H.M., Porto V.C., Lara V.S. (2016). Decreased production of proinflammatory cytokines by monocytes from individuals presenting *Candida*-associated denture stomatitis. Cytokine.

[8] Weindl G., Naglik J.R., Kaesler S., Biedermann T., Hube B., Korting H.C., Schaller M. (2007). Human epithelial cells establish direct antifungal defense through TLR4-mediated signaling. J. Clin. Investig..

[9] Zakikhany K., Naglik J.R., Schmidt-Westhausen A., Holland G., Schaller M., Hube B. (2007). In vivo transcript profiling of *Candida albicans* identifies a gene essential for interepithelial dissemination. Cell Microbiol..

[10] Lu Q., Jayatilake J.A., Samaranayake L.P., Jin L. (2006). Hyphal invasion of *Candida albicans* inhibits the expression of human beta-defensins in experimental oral candidiasis. J. Investig. Dermatol..

[11] Decanis N., Savignac K., Rouabhia M. (2009). Farnesol promotes epithelial cell defense against *Candida albicans* through Toll-like receptor 2 expression, interleukin-6 and human beta-defensin 2 production. Cytokine.

[12] Peyret-Lacombe A., Duplan H., Watts M., Charveron M., Brunel G. (2007). Antimicrobial peptide modulation in a differentiated reconstructed gingival epithelium. Cell Tissue Res..

[13] Vylkova S., Nayyar N., Li W., Edgerton M. (2007). Human beta-defensins kill *Candida albicans* in an energy-dependent and salt-sensitive manner without causing membrane disruption. Antimicrob. Agents Chemother..

[14] Vylkova S., Sun J.N., Edgerton M. (2007). The role of released ATP in killing *Candida albicans* and other extracellular microbial pathogens by cationic peptides. Purinergic Signal..

[15] Doss M., White M.R., Tecle T., Hartshorn K.L. (2010). Human defensins and LL-37 in mucosal immunity. J. Leukoc. Biol..

[16] Ferrari C.K., Souto P.C., França E.L., Honorio-França A.C. (2011). Oxidative and nitrosative stress on phagocytes function: From effective defense to immunity evasion mechanisms. Arch. Immunol. Ther. Exp..

[17] Nakai K., Kubota Y., Kosaka H. (2004). Inhibition of nuclear factor kappa B activation and inducible nitric oxide synthase transcription by prolonged exposure to high glucose in the human keratinocyte cell line HaCaT. Br. J. Dermatol..

[18] Qu X.M., Wu Z.F., Pang B.X., Jin L.Y., Qin L.Z., Wang S.L. (2016). From nitrate to nitric oxide: The role of salivary glands and oral bacteria. J. Dent. Res..

[19] Villar C.C., Zhao X.R. (2010). *Candida albicans* induces early apoptosis followed by secondary necrosis in oral epithelial cells. Mol. Oral Microbiol..

[20] Wagener J., Weindl G., de Groot P.W., de Boer A.D., Kaesler S., Thavaraj S., Bader O., Mailänder-Sanchez D., Borelli C., Weig M. (2012). Glycosylation of *Candida albicans* cell wall proteins is critical for induction of innate immune responses and apoptosis of epithelial cells. PLoS ONE.

[21] Park H., Myers C.L., Sheppard D.C., Phan Q.T., Sanchez A.A., E. Edwards J., Filler S.G. (2005). Role of the fungal Ras-protein kinase A pathway in governing epithelial cell interactions during oropharyngeal candidiasis. Cell Microbiol..

[22] Villar C.C., Chukwuedum Aniemeke J., Zhao X.R., Huynh-Ba G. (2012). Induction of apoptosis in oral epithelial cells by *Candida albicans*. Mol. Oral Microbiol..

[23] Conti H.R., Bruno V.M., Childs E.E., Daugherty S., Hunter J.P., Mengesha B.G., Saevig D.L., Hendricks M.R., Coleman B.M. (2016). IL-17 receptor signaling in oral epithelial cells is critical for protection against oropharyngeal candidiasis. Cell Host Microbe.

[24] De Carvalho Dias K., Barbugli P.A., de Patto F., Lordello V.B., de Aquino Penteado L., Medeiros A.I., Vergani C.E. (2017). Soluble factors from biofilm of *Candida albicans* and *Staphylococcus aureus* promote cell death and inflammatory response. BMC Microbiol..

[25] Baroni A., De Filippis A., Oliviero G., Fusco A., Perfetto B., Buommino E., Donnarumma G. (2018). Effect of 1064-nm Q-switched Nd: YAG laser on invasiveness and innate immune response in keratinocytes infected with *Candida albicans*. Lasers Med. Sci..

[26] Klingbeil M.F., Herson M.R., Cristo E.B., dos Santos Pinto D., Yoshito D., Mathor M.B. (2009). Comparison of two cellular harvesting methods for primary human oral culture of keratinocytes. Cell Tissue Bank..

[27] Xiao S., Zhu S., Ma B., Xia Z.F., Yang J., Wang G. (2008). A new system for cultivation of human keratinocytes on acellular dermal matrix substitute with the use of human fibroblast feeder layer. Cells Tissues Organs.

[28] Steubesand N., Kiehne K., Brunke G., Pahl R., Reiss K., Herzig K.H., Schubert S., Schreiber S., Fölsch U.R., Rosenstiel P. (2009). The expression of the beta-defensins hBD-2 and hBD-3 is differentially regulated by NF-kappaB and MAPK/AP-1 pathways in an in vitro model of *Candida* esophagitis. BMC Immunol..

[29] Feng Z., Jiang B., Chandra J., Ghannoum M., Nelson S., Weinberg A. (2005). Human beta-defensins: Differential activity against candidal species and regulation by *Candida albicans*. J. Dent. Res..

[30] Lima H.G., Pinke K.H., Gardizani T.P., Souza-Júnior D.A., Carlos D., Avila-Campos M.J., Lara V.S. (2013). Mast cells act as phagocytes against the periodontopathogen *Aggregatibacter actinomycetemcomitans*. J. Periodontol..

[31] Tang X., Yang X., Peng Y., Lin J. (2009). Protective effects of lycopene against H_2_O_2_-induced oxidative injury and apoptosis in human endothelial cells. Cardiovasc. Drugs Ther..

[32] Chan F.K., Moriwaki K., De Rosa M.J. (2013). Detection of necrosis by release of lactate dehydrogenase activity. Methods Mol. Biol..

[33] Elahi S., Pang G., Ashman R.B., Clancy R. (2001). Nitric oxide-enhanced resistance to oral candidiasis. Immunology.

[34] Campisi G., Panzarella V., Giuliani M., Lajolo C., Di Fede O., Falaschini S., Di Liberto C., Scully C., Lo Muzio L. (2007). Human papillomavirus: Its identity and controversial role in oral oncogenesis, premalignant and malignant lesions (review). Int. J. Oncol..

[35] Velicescu M., Yu J., Herbert B.S., Shay J.W., Granada E., Dubeau L. (2003). Aneuploidy and telomere attrition are independent determinants of crisis in SV40-transformed epithelial cells. Cancer Res..

[36] Kibe T., Kishida M., Kamino M., Iijima M., Chen L., Habu M., Miyawaki A., Hijioka H., Nakamura N., Kiyono T. (2011). Immortalization and characterization of normal oral epithelial cells without using HPV and SV40 genes. Oral Sci. Int..

[37] Narisawa-Saito M., Kiyono T. (2007). Basic mechanisms of high-risk human papillomavirus-induced carcinogenesis: Role of E6 and E7 proteins. Cancer Sci..

[38] Wilson D., Thewes S., Zakikhany K., Fradin C., Albrecht A., Almeida R., Brunke S., Grosse K., Martin R., Mayer F. (2009). Identifying infection-associated genes of *Candida albicans* in the postgenomic era. FEMS Yeast Res..

[39] Naglik J.R., Moyes D., Makawana J., Kanzaria P., Tsichlaki E., Weindl G., Tappuni A.R., Rodgers C.A., Woodman A.J., Challacombe S.J. (2017). Quantitative expression of the *Candida albicans* secreted aspartyl proteinase gene family in human oral and vaginal candidiasis. Microbiology.

[40] Gasparoto T.H., Gaziri L.C., Burger E., de Almeida R.S., Felipe I. (2004). Apoptosis of phagocytic cells induced by *Candida albicans* and production of IL-10. FEMS Immunol. Med. Microbiol..

[41] Merighi S., Gessi S., Varani K., Fazzi D., Borea P.A. (2012). Hydrogen sulfide modulates the release of nitric oxide and VEGF in human keratinocytes. Pharmacol. Res..

[42] Hussain R., Oliynyk I., Roomans G.M., Bjorkqvist M. (2013). Modulation of ENaC, CFTR, and iNOS expression in bronchial epithelial cells after stimulation with *Staphylococcus epidermidis* (94B080) and *Staphylococcus aureus* (90B083). APMIS.

[43] Rouabhia M., Mukherjee P.K., Lattif A.A., Curt S., Chandra J., Ghannoum M.A. (2011). Disruption of sphingolipid biosynthetic gene IPT1 reduces *Candida albicans* adhesion and prevents activation of human gingival epithelial cell innate immune defense. Med. Mycol..

[44] Weindl G., Wagener J., Schaller M. (2011). Interaction of the mucosal barrier with accessory immune cells during fungal infection. Int. J. Med. Microbiol..

[45] Collette J.R., Zhou H., Lorenz M.C. (2014). *Candida albicans* suppresses nitric oxide generation from macrophages via a secreted molecule. PLoS ONE.

[46] Dale B.A., Kimball J.R., Krisanaprakornkit S., Roberts F., Robinovitch M., O’Neal R., Valore E.V., Ganz T., Anderson G.M., Weinberg A. (2001). Localized antimicrobial peptide expression in human gingiva. J. Periodontal Res..

[47] Feger F., Varadaradjalou S., Gao Z., Abraham S.N., Arock M. (2002). The role of mast cells in host defense and their subversion by bacterial pathogens. Trends Immunol..

